# The connecting adolescents to reduce relapse (CARR) trial: study protocol for a randomized controlled trial comparing the efficacy of Groups 4 Health and cognitive behaviour therapy in young people

**DOI:** 10.1186/s12889-019-7011-y

**Published:** 2019-06-20

**Authors:** Tegan Cruwys, Catherine Haslam, Zoe C. Walter, Joanne Rathbone, Elyse Williams

**Affiliations:** 1 Research School of Psychology, The Australian National University, Canberra, ACT 2601 Australia; 2 School of Psychology, University of Queensland, Brisbane, 4072 QLD Australia

**Keywords:** Social isolation, Loneliness, Group psychotherapy, Mood disorders, Depression, Mental health, Adolescence, Youth, Social identity

## Abstract

**Background:**

Depression is the leading cause of disability in young people (aged 15–25) globally. Loneliness is a major factor in the development and relapse of depression in young people, yet few interventions directly address loneliness. Groups 4 Health (G4H) – a novel, theoretically derived group psychotherapy intervention – may address this disconnect. Previous trials (Phase I and Phase II) have found G4H to be efficacious in reducing symptoms of depression. However, the efficacy of G4H compared to current evidence-based treatments (Phase III) has not been investigated. This protocol details the design and methodology of the Connecting Adolescents to Reduce Relapse (CARR) trial, a randomised control trial assessing the efficacy of G4H in young people relative to cognitive behavioural therapy (CBT).

**Methods:**

The CARR trial is a two-arm non-inferiority randomised controlled trial that will compare the efficacy of G4H to the most widely used evidence-based treatment for depression, CBT, at program completion and 6- and 12-month follow up. Participants will be 200 young people (aged 15–25) with symptoms of depression and/or loneliness recruited from community and university mental health services. We hypothesise that the interventions will be comparable in reducing depression symptoms, but that G4H will be superior in reducing loneliness. Because loneliness is a primary risk factor for depression relapse in young people, we therefore expect the benefits of Groups 4 Health to be particularly apparent at 12-month follow up.

**Discussion:**

This trial will be the first to evaluate an intervention that targets loneliness, in comparison to the current gold standard treatment approach – CBT. If found to be effective, this program offers a new approach to treatment and relapse prevention of depression among young people.

**Trial registration:**

Trial prospectively registered on ANZCTR (ACTRN12618000440224), registered on 27/03/2018.

**Electronic supplementary material:**

The online version of this article (10.1186/s12889-019-7011-y) contains supplementary material, which is available to authorized users.

## Background

Depression has been ranked as the single greatest cause of disability in young people aged 15–25 [1]. Although both pharmacological and psychotherapy treatments are effective in reducing acute symptoms in young people [2], they come with a range of downsides. The cost of these treatments can be prohibitive [3], particularly because depression is more prevalent among disadvantaged communities [4]. There is also a global shortfall of mental health professionals who have the level of training required to provide these evidence-based treatments. This shortfall is most apparent in areas of greatest need, such as in non-urban areas with high rates of socioeconomic deprivation [5].

There are other barriers to the effective treatment of depression, particularly for young people. Previous research suggests that only a minority of young people with depression speak to a health professional about it [6], and only a minority of those who do receive best-practice treatment [2]. For instance, a common pathway to treatment is antidepressant medication prescribed following a consultation with a General Practitioner. However, the most common antidepressants prescribed for adults are not suitable for young people due to elevated suicide risk [7]. Even for those medications that are safe for adolescents, compliance rates are as low as 45% [8], often attributed to side effects such as weight gain and drowsiness. While most young people prefer non-drug treatment [9], the stigma associated with therapy often leads to treatment avoidance [10]. For these reasons, there is a need to develop treatment alternatives for young people with depression that are cost-effective, non-stigmatising, and widely accessible.

Furthermore, although short-term treatments are generally effective at alleviating acute symptoms, existing approaches to relapse prevention are only minimally effective. About 80% of individuals who have experienced a depressive episode relapse, experiencing an average of four episodes across a lifetime [11]. Even among those who receive best practice treatment, approximately one-third relapse within 18 months [12]. Therefore, new innovations in treatment should prioritise reducing depression recurrence.

### Loneliness as a promising target for intervention

Research has demonstrated that loneliness both precipitates and maintains depression. Longitudinal studies have shown that loneliness is a powerful predictor of depression onset, even after controlling for other candidate variables [13]. Loneliness also predicts poorer outcomes in depression treatment [14] and increases the risk of relapse [15]. These findings suggest that targeting loneliness may offer an effective approach to preventing relapse. Research has shown that when a person with depression joins a group, they reduce their risk of relapse four years later by approximately 24% [16].

Several diverse approaches to targeting loneliness have been developed [17]. These approaches mostly fall into two broad categories: either (1) *befriending* interventions, which seek to increase opportunities for social contact or provide social support, or (2) *cognitive* interventions which seek to address maladaptive cognitions or inadequate social skills. However, most of these interventions are not informed by theory, and evidence of their effectiveness is mixed [18, 19]. Indeed, Masi and colleagues stated that “﻿loneliness interventions to date have not attained the degree of efficacy achieved by interventions targeting other social and behavioural outcomes” ([19], p.257). Furthermore, loneliness interventions have, by and large, been developed and evaluated for older adults, with little attention paid to young people. Given the evidence that adolescents and young adults report some of the highest levels of loneliness in the population [20], there is particular need for research in this area.

### Groups 4 health

Groups 4 Health (G4H) was developed to fill this void as a theory-driven, group psychotherapy intervention that targets loneliness [21, 22]. G4H is informed by the Social Identity Approach to Health (see [23, 24] for a summary), which is a conceptual framework that articulates how social group memberships, and the social identities associated with them, affect health and wellbeing.

To date, two trials of G4H have been completed. The first was a Phase I pilot [25], in which the program was delivered to 81 people (*M*_age_ = 20.95; *SD* = 5.05) presenting with loneliness and affective disturbance (symptoms of depression or anxiety), of whom 54 completed G4H, and 26 completed the 6-month follow up. This group was compared to a matched, but not randomised, assessment-only control group. G4H was found to significantly improve mental health (depression, general anxiety, social anxiety, and stress), well-being (self-esteem, life satisfaction) and social connectedness (loneliness, social functioning), both on program completion and at 6-month follow up. Moreover, G4H recipients had significantly better outcomes than those in the control group at the 6-month follow up on measures of depression, anxiety, stress and self-esteem. In line with the theorised mechanisms of the G4H intervention, analyses also showed that participants whose identification with their G4H group and their subjective sense of belonging to multiple groups increased were most likely to experience improvements in depression, anxiety, stress, loneliness, and life satisfaction.

The second trial was a Phase II randomised control trial (RCT; [23]) in a sample of adults recruited from the community (*M*_age_ = 31.06; *SD* = 12.80). This RCT examined the efficacy of G4H compared to a Treatment-As-Usual (TAU) control among people with current depression symptoms or health-professional diagnosis of a mental illness (*N* = 120). In this trial, the TAU group received a diverse range of interventions, with more than 50% receiving antidepressant medication, psychotherapy, or both. Intention-to-treat analyses showed that people in the G4H condition experienced a significant decline in loneliness, social anxiety and primary care attendance, and a significant increase in their sense of belonging to multiple groups, relative to TAU. There was also evidence of significant pre-post improvement in depression among those who completed G4H, although this was not significantly greater than the improvement seen in the TAU condition.

These data suggest that G4H has good potential to benefit the mental health of young people in treatment. The next important stage is to test G4H against a *uniform, active treatment.* This is the purpose of the present program of research with young adults with depression.

### Aims

The project aims to evaluate G4H in comparison to the current gold standard evidence-based psychological treatment for youth depression, CBT. Based on the existing evidence, we predict:

- *Hypothesis 1:* At program completion, G4H will be superior to CBT in reducing loneliness (a major risk factor for relapse in depression).
- *Hypothesis 2:* At program completion, G4H will be as effective as CBT in reducing symptoms of depression (i.e., be non-inferior)
- *Hypothesis 3:* The benefits of G4H for loneliness and depression will be sustained at 12-month follow up.

## Method

### Trial design

CARR is a two-arm non-inferiority parallel (1:1 ratio) randomised controlled trial. A summary of this study’s compliance with Standard Protocol Items: Recommendations for Interventional Trials (SPIRIT), is provided in the Additional file 1.

### Participants

Participants will be 200 young people presenting with psychological distress recruited from two sources in South East Queensland, Australia: (1) a community youth mental health service (headspace, five sites), and (2) a university psychology clinic (University of Queensland). The following criteria will be used in recruitment:

#### Inclusion

- Aged between 15 to 25 years
- English speaking
- The presence of depressive symptoms on the PHQ-9 (> = 5; corresponding to at least the mildly impairing clinical range; [23]);
  OR
- a health-professional diagnosed mental health condition (e.g., major depressive disorder);
  OR
- The presence of elevated loneliness on the UCLA (> = 40; approximately 1 SD above the mean for adolescents; [26]).

#### Exclusion

- Currently in receipt of other evidence-based treatment for depression (psychotherapy or psychopharmacology)
- Current high risk suicidal ideation or severe self-harming behaviour.
- Current psychotic episode or severe personality disorder that would interfere with ability to participate in group work.
- Severe neurological condition or severe learning impairment that would hinder engagement with the content of the proposed interventions
- Current alcohol or other substance dependence / intoxication

Participants will be monitored throughout the trial for any change in these inclusion and exclusion criteria (e.g., in adjunct treatment status, or in suicide risk). Participants whose mental health declines for any reason will be provided with additional one-on-one supportive counselling by their group facilitator and referred to additional treatment where indicated. If a person who is initially eligible for the trial subsequently meets an exclusion criterion at any point during the active-treatment phase of the trial (T0-T5) they will be excluded from per-protocol analyses, but not intention-to-treat analyses.

### Recruitment

Allied health professionals and professionals-in-training at the two services will be invited to participate in an information session, which will include selection criteria for the participants, and a flyer for them to display within the service to inform participants of the program. These professionals will identify eligible patients for inclusion into the study on a rolling basis. They will approach potential participants and provide them with contact details for trial staff, including a website with links to the Participant Information Sheet and Consent Form. Research staff will contact potential participants to conduct screening and assessment, explain the study in more detail, answer any questions arising, and go through the consent process. If a potential participant indicates thoughts of self-harm in the baseline measures (i.e., a score of 3 or 4 on item 9 of the PHQ-9), a risk assessment will be conducted at this stage. Participants will provide fully informed, written and voluntary consent. Additionally, for those participants aged 16 or under, their parent or guardian will be informed of their participation and provided with an information sheet with the permission of the young person. However, primacy will be given to the consent of the young person, in line with the organisational policies of the research setting (which does not require parental consent for young people to access mental health services). Participants will be eligible to complete the CARR trial either as an (evidence-based) alternative to standard care, or while they are on a waiting list for standard care.

#### Incentives

Participants will not be offered incentives to undertake the intervention, other than receipt of free, evidence-based mental health care. Those participants who are also students enrolled in first-year psychology courses will receive course credit for their participation in the program. To improve attendance at Module 5, where post-program assessments are conducted, all participants will be offered either a coffee or supermarket voucher (AUD$15 value) for attendance. To minimise attrition, participants will be offered AUD$50 incentive to complete each of the 6-month and 12-month follow up assessments. Participants will be followed up unless they actively withdraw from the trial (i.e., data will be sought from participants who did not attend group sessions or who no longer meet inclusion criteria, in order to honour the intention-to-treat principle).

### Randomisation

Participants who are eligible will be randomly assigned by the research team following simple randomisation procedures (computerized random numbers using Excel random number generator feature) to either G4H or CBT using a 1:1 ratio. Participants will be randomised at the group level once sufficient participants have been recruited for a group (minimum of 5). That is, participants will first be recruited into a treatment group, and each group will then be randomised to receive either G4H or CBT, using the randomisation process outlined above. Neither the participant nor the research team will be blind to intervention status. Assessment of primary outcomes at each timepoint will occur via a web interface to ensure that this is not affected by intervention status.

### Intervention

Participants will be randomly assigned to receive either G4H or CBT. Groups will not commence unless a minimum of 5 people have consented to participate, with the maximum number of people recruited to each group being 9 people. Interventions are matched for contact time and mode of delivery, and both include five group sessions of 60–90 min in duration, run weekly for the first four weeks, and a final “booster” session that is held one month later. Postgraduate students in professional psychology (with provisional registration) will facilitate the groups (2 facilitators per group). Treatment fidelity and consistency of program delivery will be maintained by providing: a half-day of training, weekly group supervision from a registered clinical psychologist, direct observation by investigators to ensure quality, and regular team meetings with the research team. Additionally, G4H facilitators will anonymously complete a brief questionnaire at the end of each session to assess (a) what session activities were covered, (b) the length of session, and (c) facilitator session satisfaction and ease of delivery. Facilitators will also record participant attendance.

#### Groups 4 health (G4H)

**Fig. 1:**
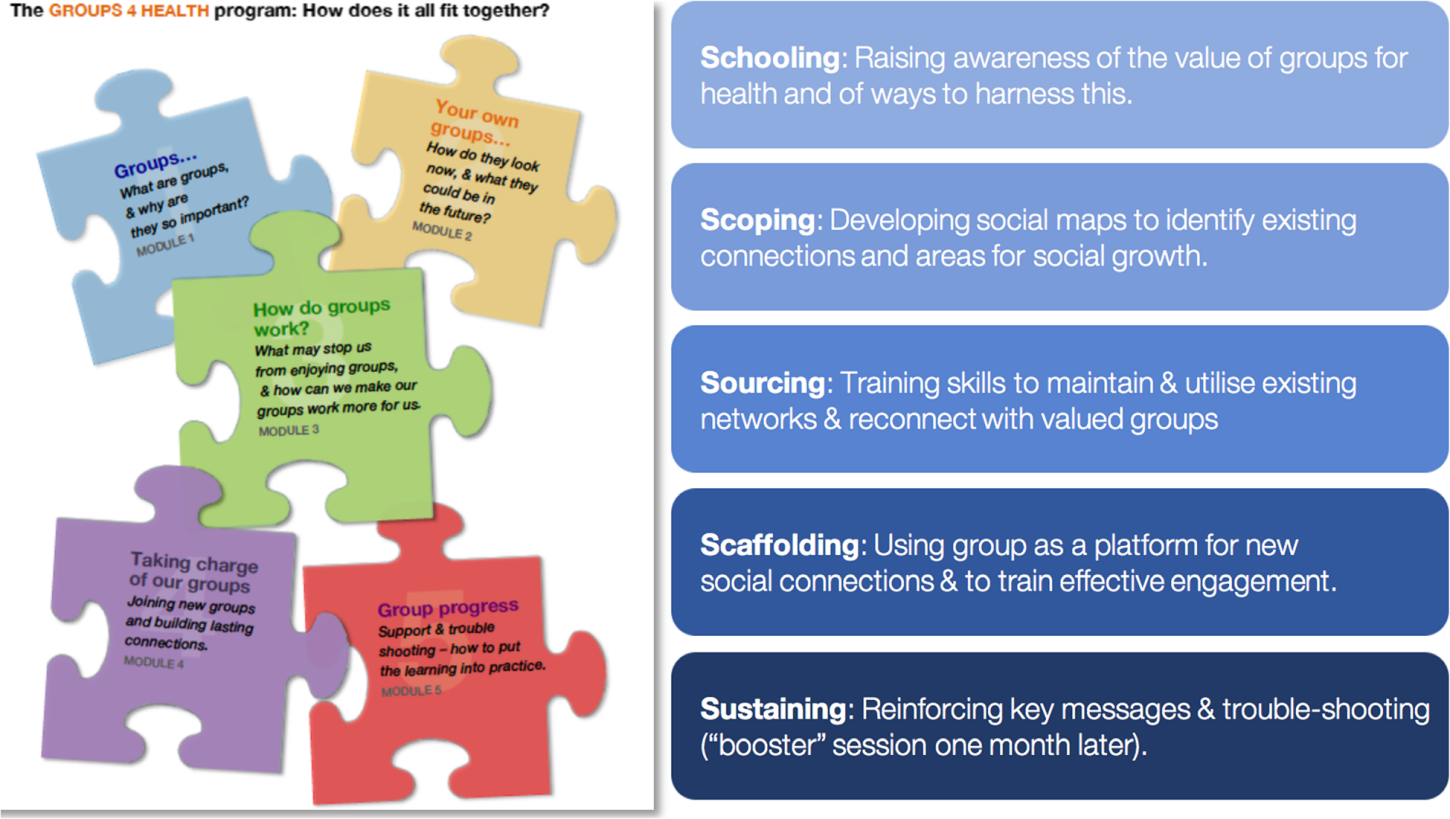
Overview of the Groups 4 Health Modules

G4H is a manualised, five-session group-based intervention designed to reduce loneliness through facilitating social group integration [21, 22]. The program is comprised of five modules — the contents of which are summarised in Fig. 1 below.

##### Module 1: Schooling

This module seeks to raise awareness of the beneficial effects that social group memberships have for health. Module 1 highlights the costs of ignoring the social dimensions of health and points out that using all of the social resources at our disposal generally leads to optimal health outcomes. The module also emphasises people’s power to counter these effects by learning how best to develop, maintain and harness group-based resources.

##### Module 2: Scoping

Explores each person’s current group-based resources. This module engages participants in the process of *social identity mapping* [25]. This tool was developed to explore respondents’ social identities in a way that is engaging, visual, and concrete. The map is then used to facilitate a discussion of how people would ideally like their groups to look in the future and identify any gaps in group networks.

##### Module 3: Sourcing

Targets development of strategies needed to identify and strengthen existing valued social identities. The goal of Module 3 is to assist people to optimize and sustain their group memberships over the long term. Participants develop solutions to barriers to making the most of existing groups.

##### Module 4: Scaffolding

Uses the G4H group as an exemplar for developing and embedding new social group connections, whilst also identifying which connections to develop. Group members are facilitated to prepare a social plan of action. These social plans are then trialled between this and the final module, which takes place one month later.

##### Module 5: Sustaining

Is a booster session, held one month after Module 4 that seeks to troubleshoot any problems that people have encountered in implementing their social plans. Module 5 also revisits social identity maps, created in Module 2, to see how they have changed over the course of the program. The skills that have been learned across the previous modules are reviewed and key messages reinforced with the goal of encouraging long-term maintenance.

The program is supported by a facilitator manual and client workbook. The workbook contains a summary of the key learnings for each session, with sufficient space to complete activities (both within the sessions and as homework) and document any relevant notes and plans to achieve a person’s individual goals.

#### Group cognitive behaviour therapy

The active comparison condition will involve the delivery of the current best-practice intervention for the treatment of adolescent depression: CBT. Specifically, an existing brief manualised program for group-based CBT for depression [27] will be used with minor adaptations to ensure comparable structure and amount of face-to-face contact as the G4H condition. This specific manualised version of CBT has been found to be effective in reducing depression symptoms in a sample of high-risk adolescents relative to active control groups (*N* = 341; [28]). Indeed, a meta-analysis found that this particular manualised program showed greater effect sizes than similar interventions with longer duration [29].

The manualised program involves the following components: (1) psychoeducation, in which participants learn about the link between thoughts, mood, and behaviour; (2) behavioural activation, in which participants learn strategies to increase activities that boost their sense of pleasure and achievement; and (3) cognitive restructuring, in which participants learn strategies for challenging and reframing unhelpful thinking patterns that maintain depression. The program also includes homework tasks and group-based activities.

### Measures

Each participant will be asked to complete online surveys (or paper-and-pencil if they prefer) at eight time points. The full questionnaire package measuring all outcomes of interest will be administered before the intervention (T0), on its completion (T5), 6 months later (T6) and 12 months later (T7) – see Table 1 for a summary. These should take about 10–20 min on each occasion. In addition, a brief one-page questionnaire will be administered at the end of each session (T1, T2, T3, and T4), which will (only) include measures of depression symptoms, loneliness, module feedback, and group identification.

**Table 1:** Overview of CARR measures and timepoints of measurement

Construct	Measure	T0 Baseline	T1 After session 1	T2 After session 2	T3 After session 3	T4 After session 4	T5 Post-treatment	T6 6-month follow up	T7 12-month follow up
Primary outcomes	Depression (DASS-21)	•	•	•	•	•	•	•	•
	Depression (PHQ-9)	•					•	•	•
	Loneliness (4 item)	•	•	•	•	•	•	•	•
	Loneliness (20 item)	•					•	•	•
Secondary outcomes	Social anxiety	•					•	•	•
	GP attendance (last month)	•					•	•	•
	Wellbeing	•					•	•	•
	Life satisfaction	•					•	•	•
	Self esteem	•					•	•	•
	Subjective physical health	•					•	•	•
Process measures	Multiple group membership	•					•	•	•
	Multiple group compatibility	•					•	•	•
	Mental illness identity	•					•	•	•
	Group cohesion						•		
	Therapy group identification		•	•	•	•	•		
	Service identification						•		
Acceptability/feasibility	Useful		•	•	•	•	•	•	•
	Enjoyed						•	•	•
	Interesting						•	•	•
	Learnt something						•	•	•
	Homework completion		•	•	•	•	•		
	Facilitator questionnaire		•	•	•	•	•		
	Attendance		•	•	•	•	•		

Notes: *DASS21* Depression Anxiety Stress Scales (21 item)

*PHQ-9* Patient Health Questionnaire (9 item).

*GP* General Practitioner

### Primary outcomes

*Depression, Anxiety and Stress Scale* [30]*.* This measure comprises 21 items, 7 for each construct, for example “I felt that life was meaningless”, rated on a 4-point scale (0 = did not apply to me at all, 3 = applied to me very much, or most of the time. It has been validated for use with adolescents as young as 11 years in Australia [31], with an improvement greater than 3 points considered clinically significant [32]. The full DASS-21 (including anxiety and stress subscales) will be administered at T0, T5, T6 and T7, while the depression subscale will be administered at all timepoints T0-T7. The 9-item Patient Health Questionnaire is included as an additional measure of depression (T0, T5, T6, T7); this will be used as a screening tool rather than as the primary outcome measure.

*UCLA-20:* The UCLA Loneliness scale has been validated for use with a young adult sample [33, 34]. It comprises 20 items, for example “Felt isolated from others” requiring a response on a 4-point scale (1 = never, 4 = often). The full scale will be included at T0, T5, T6 and T7, and a 4-item version of the UCLA scale will be included in the end of session questionnaire (T1-T4).

### Secondary outcomes

#### Social anxiety

This was assessed with the Social Phobia Inventory (SPIN; [35]), comprising 17 items (e.g., “Being embarrassed or looking stupid are among my worst fears”). Each item is rated on a 5-point scale (1 = not at all, 5 = extremely), with higher scores indicating greater social anxiety.

#### General practitioner (GP) visits

This was measured with a single item: “How many times have you been to see a general practitioner (medical doctor) in the last month” (as used in [25, 36]).

#### Subjective wellbeing

The Short Warwick Edinburgh Mental Wellbeing Scale [37] will be used to index well-being. It is a 7 item scale with positively worded items (e.g., “I’ve been thinking clearly”), which respondents rate for how often they have experienced them over the past two weeks, on a 5-point scale (1 = none of the time; to 5 = all of the time).

#### Life satisfaction

The Satisfaction With Life Scale (SWLS; [38]) will be included to assess global life satisfaction. Participants will respond to 5 items (e.g., “I am satisfied with my life”) on a 7-point scale (1 = strongly disagree, 7-strongly agree).

#### Self esteem

This was measured using the Single Item Self Esteem measure (SISE; [39]). Participants responded to the item “I have high self esteem” on a 4-point scale (1 = not at all true of me, 4 = very true of me).

#### Subjective physical health

Participant’s self-rated health status was measured using a single item “My current overall health is…”, with responses ranging from 1 (very poor) to 7 (very good). This measure is widely used in community and population health studies and has been found to be a valid predictor of physical health and mortality risk [40].

### Process measures

Five measures will be used to index different aspects of social identification and group processes as follows:

(i). *Social identification with treatment group:* This is a 4-item scale [41] was used to assess identification with the treatment group (e.g., “I identify with my G4H group/CBT group”) and is rated on a 7-point scale (1 = strongly disagree, 7 = strongly agree).
(ii). *Social identification with service:* This is a 4-item scale [41] was used to assess identification with the service (e.g., “I identify with Headspace/the UQ Psychology Clinic”) and is rated on the same 7-point scale used above (1 = strongly disagree, 7 = strongly agree).
(iii). *Multiple group membership.* This will be measured using the Multiple Group Membership scale from the Exeter Identity and Transition Scales [42]. The 3-item version of the scale was used to index people’s strength of connectedness to multiple groups [43]. Participants are asked to rate each item (e.g., “I am a member of lots of different social groups”) on a 5-point scale (1 = do not agree at all, 5 = agree completely).
(iv). *Social identity centrality:* This is a 7-item scale adapted from [44] assessing the psychological salience of a particular social category membership, here as someone with mental health problems (e.g., “I often think about the fact that I am a person who has problems with mental health”; 1 = do not agree at all, 5 = agree completely).
(v). *Group Cohesion.* The Group Environment Scale – Cohesion subscale [45] will be used to measure treatment group cohesion. Nine items (e.g., “There is a strong feeling of belongingness in this group”) are measured on a 7-point scale (1 = not at all, 7 = very much).

### Acceptability and feasibility

Participants will be asked to rate their perceived usefulness of the program after every session (T1-T5): “How useful did you find today’s module?” on a scale from 1 (not useful at all) to 10 (very useful). At T5, T6, and T7, participants will additionally be asked to rate how much they enjoyed the program, found the program interesting, learnt from the program, and how likely they were to use the techniques they learned during the program (each on a 1–10 scale). Facilitators will also provide a rating of the perceived usefulness, relevance, learning, and enjoyment for participants after each module (T1-T5).

Qualitative feedback on the program will be sought from participants and facilitators, both in the form of written feedback (at T1-T7) as well as in a series of focus groups conducted separately with a subgroup of participants (including those who withdrew from the program) and facilitators.

### Sample size power calculations

**Fig. 2:**
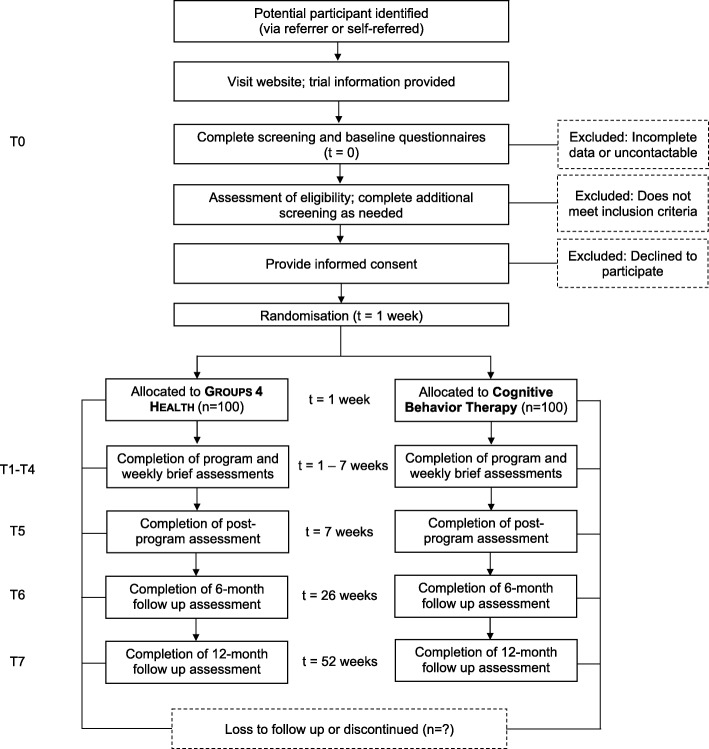
Flow of participants in the CARR trial

The CARR sample size was determined by power calculations for a non-inferiority trial, using effect sizes and retention rates from our published pilot study. We have estimated the non-inferiority margin based on historical evidence for the effect size of the active comparator (based on recommendations by [46] and [47]). The effect size of this CBT program versus control was *d* = 0.49 at 6 month follow up (recalculated from [28] using methods recommended by [19]). This corresponds to 2.2 points on the DASS-21 depression subscale (using the expected standard deviation for an adolescent population of 4.53; [31]). To have 0.80 power to detect this difference with an alpha level of 0.05*,* 67 people per condition would need to be retained at follow up. Assuming 50% eligibility and 67% survival rates, which are consistent with our pilot data, we anticipate that screening 400 people for eligibility (see Fig. 2) will lead to retention of *N* = 67 per condition at follow up [48]. However, this is a conservative estimate of retention due to the addition of incentives for follow up data completion in this trial [49].

### Analyses

Data quality will be improved by using a web interface that prompts (but does not force) participants to complete missing items. This will reduce missing data and prevent errors from manual data entry. Data quality will be checked by assessing ranges for each variable and interrogating outliers. We will examine the differences in baseline characteristics between the groups descriptively. Since differences between randomised groups at baseline could have occurred by chance, no formal significance testing will be conducted (in accordance with recommendations by [50]). No covariates will be included in the primary analysis (in accordance with recommendations by [51]). However, we will include exploratory analyses which assess the interaction between condition and baseline characteristics where indicated.

To assess the effect of intervention, group differences on the primary and secondary outcome measures, with all available timepoints included in the model, will be examined using a series of mixed effects repeated measures (MMRM) models, which will specify timepoint, participant, and therapy group as levels in the analyses. MMRM is a full information maximisation likelihood estimation strategy that can model data even when some observations are missing, and thus honours the intention-to-treat principle [52]. For all hypothesis testing, both intention-to-treat analyses (ITT; where all available data are included in MMRM) and per protocol analyses (PP; only including participants who met eligibility criteria throughout the trial period, were randomly assigned, completed the intervention according to the protocol, and had both baseline and follow up data) will be completed. Best fit, as determined by Information Criteria (e.g. AIC; BIC) method will be used for determining the covariance structure for MMRM models.

H1 will be considered supported if there is evidence of significant improvements in loneliness in the G4H condition (H1a), and a significant condition by time interaction, such that improvements in loneliness over time will be greater for the G4H condition than in the CBT condition (H1b). H2 will be considered supported if there is evidence of significant improvements in depression in both conditions, with a non-significant condition by time interaction. The non-inferiority margin will be a DASS-21 depression change score difference of 2.20. H3 will be considered supported if there is no significant increase in depression and loneliness scores between T6 and T7 in the G4H condition.

## Discussion

Depression is a leading cause of disability among young people. Although there are currently evidence-based pharmacological and non-pharmacological treatments for depression, young people face barriers to treatment, including side effects, cost, accessibility, and stigma. Additionally, relapse after treatment is common [12].

Given this, it is important to develop interventions that are accessible to young people and target primary causes of depression and relapse. Loneliness is a primary cause of depression onset and relapse in young people [53]. The current protocol outlines a non-inferiority RCT to assess the efficacy of a novel intervention that targets loneliness in young people, compared to the current gold standard evidence-based psychological treatment for depression (CBT). If found to be effective, this program offers a new approach to treatment and relapse prevention of depression among young people that is cost-effective, accessible, and destigmatising.

## Additional file

Additional file 1: SPIRIT 2013 Checklist: Recommended items to address in a clinical trial protocol and related documents*. (PDF 85 kb)

## Data Availability

Data sharing is not applicable to this article as no datasets were generated or analysed in the preparation of the trial protocol.

## Abbreviations

AIC: Akaike Information Criterion
ANZCTR: Australian New Zealand Clinical Trials Registry
BIC: Bayes Information Criterion
CARR: Connecting Adolescents to Reduce Relapse
CBT: Cognitive Behaviour Therapy
DASS21: Depression, Anxiety, Stress Scales – 21 item
G4H: Groups 4 Health
ITT: Intention to treat
MMRM: Mixed model repeated measures
NHMRC: National Health and Medical Research Council
PHQ-9: Patient Health Questionnaire – 9 item depression scale
PP: Per protocol
RCT: Randomised Control Trial
